# 

**Published:** 1849-07

**Authors:** 


### CONTENTS

### OF NO. I.

---

### ORIGINAL COMMUNICATIONS.

### ESSAYS AND CASES.

ART. I.—Cholera, as it appeared in Clarksville, Tennessee; with Reflections. By Dr. E. B. HASKINS ....................1

ART. II.—Two Cases of Nephritis produced by over doses of Balsam Copaiba. By DAVID W. YANDELL, M.D., of Louisville, Kentucky....................15

ART. III.—Cases of Thoracic Disease. By Dr. THOMAS C. OSBORNE, of Erie, Alabama....................23

ART. IV.—A Case of Congenital Dropsy of the Cranial, Thoracic, and Abdominal Cavities, complicated with Spina-Bifida, impeding labor, and rendering perforation of the head necessary to accomplishing delivery. By Dr. THOS. LIPSCOMB, of Shelbyville, Tenn....................34

### REVIEWS.

ART. V.—Obstetrics—the Science and the Art. By CHARLES D. MEIGS, M.D., Professor of Midwifery and the Diseases of Women and Children, in the Jefferson Medical College, one of the Physicians to the Lying-in Department of the Pennsylvania Hospital, Vice President of the Philadelphia College of Physicians, Member of the American Philosophical Society, of the American Medical Association, etc., etc. With one hundred and twenty illustrations....................37

SELECTIONS FROM AMERICAN AND FOREIGN JOURNALS.

Spina-Bifida Cured by Injections of Iodine .......................61
Origin of Sugar in the Animal Economy.......................61
Uses of the Pancreatic Juice in Digestion.......................62
On the Treatment of Internal Strangulation of the Intestines by Strychnine.......................62
The Heart Clot—its Cause.......................63
On Prolapsus Uteri, by Prof. Hohl .......................75
On the Treatment of Chronic Inflammation of the Bladder by Injections of Nitrate of Silver, by R. L. McDonnell, Esq., M. D.......................77
Observations on a mode of Annihilating the Pains which follow Surgical Operations, by Jules Roux.......................80
On Cod-liver Oil in Phthisis, by Dr. C. J. B. Williams .......................83

ORIGINAL INTELLIGENCE.

Resignation of Professor Short.......................85
Professor of Materia Medica in the University of Louisville.......................86
Ozone.......................87
Death of Prof. Barbour .......................89
Progress of Cholera.......................89
Ohio Medical College .......................91
Transylvania University.......................92
Books received.......................92

